# E-therapies in England for stress, anxiety or depression: what is being used in the NHS? A survey of mental health services

**DOI:** 10.1136/bmjopen-2016-014844

**Published:** 2017-01-23

**Authors:** M R Bennion, G Hardy, R K Moore, A Millings

**Affiliations:** 1 Department of Psychology, University of Sheffield, Sheffield, UK; 2 Department of Computer Science, University of Sheffield, Sheffield, UK

**Keywords:** NHS, e-therapies, England, depression, anxiety, stress

## Abstract

**Objective:**

To document the range of web and smartphone apps used and recommended for stress, anxiety or depression by the National Health Service (NHS) in England.

**Design:**

The study was conducted using Freedom of Information (FOI) requests and systematic website searches.

**Data sources:**

Data were collected via FOI requests to NHS services between 13 February 2015 and 31 March 2015, and searches conducted on NHS apps library websites between 26 March 2015 and 2 November 2015.

**Data collection/extraction methods:**

Data were compiled from responses to: (1) FOI requests sent to all Improving Access to Psychological Therapies (IAPT) services and NHS Mental Health Trusts in England and (2) NHS apps library search results.

**Results:**

A total of 61 (54.95%) out of the then 111 IAPT service providers responded, accounting for 191 IAPT services, and all 51 of the then NHS Mental Health Trusts responded. The results were that 13 different web apps and 35 different smartphone apps for depression, anxiety or stress were available through either referral services or the online NHS Apps Libraries. The apps used and recommended vary by area and by point of access (online library/IAPT/trust).

**Conclusions:**

Future research is required to establish the evidence base for the apps that are being used in the NHS in England. There is a need for service provision to be based on evidence and established guidelines.

**Strengths and limitations of this study**

- We present the first comprehensive list of e-therapies used and recommended for common mental health problems across the National Health Service in England, gathered through systematic means.
- Freedom of Information (FOI) requests rely on the expertise of those responsible with handling FOI requests in any given organisation. FOI responses may have varied in their degree of thoroughness, and this information was not always available to the research team.
- Some respondents gave overarching detail of their provider's e-therapy provisions, while other responses were broken down at the level of individual Improving Access to Psychological Therapies (IAPT) services hosted by a given IAPT provider.

## Introduction

The combination of increased demand and financial pressures has forced health services to explore new and innovative methods of delivery at minimum cost. The internet and connected devices offer one potential solution to this challenge, which governments have begun to recognise, encouraging the use of digital services (see Australia's digital hospital^1^) and internet mental health services in Norway and Sweden.^2^ However, it is unclear to what extent these initial steps are exploiting the digital potential in some countries. In the UK, according to a survey published in 2014, only 2% of the population reported any digitally enabled transaction with the National Health Service (NHS) despite an estimated 59% of the UK citizens possessing a smartphone and 84% of adults using the internet.^3^ In England, the underuse of digital platforms in the NHS has been recognised by the publication of a 5-year plan to reshape care delivery and use technology in the delivery of all kinds of healthcare.^4^

The current paper focuses on e-therapy in England, where the landscape of digital mental health service provision is not well delineated. This can be attributed to several factors: inadequate reporting; changing service recommendations; nationwide reorganisations of service provision infrastructure; and the rapid development and growth of the digital sphere itself. What is clear, though, is the increasing need for such services: a 2014 survey suggested that one in 10 people in England wait more than a year for mental health assessment,^5^ and in the UK as a whole, it is estimated that by 2030 there will be 2 million more adults with mental health problems.^6^ E-therapy has the potential to reduce waiting lists, make treatment more cost-effective, reduce the time and expense of travel, stimulate self-management^7^ ^8^ and decrease the workload of mental health professionals.^9^ ^10^

The current study is based on requests made under the provisions of the UK Freedom of Information Act 2000 in 2015, and systematic enquiries on NHS websites. Under the Freedom of Information (FOI) act, publicly funded bodies are obliged to respond to requests for certain information from members of the public. The resultant data document the current state of digital mental health service provision in England, identifying what e-therapies are used and recommended across the NHS.

There are multiple ways in which e-therapies have been defined and categorised in the literature. Riper *et al*^11^ describes e-mental health as ‘the use of information and communication technology (ICT)—in particular the many technologies related to the Internet—when these technologies are used to support and improve mental health conditions and mental health care’. Other researchers have categorised e-therapies according to the amount of therapist support in them,^12^ or the exact manner in which the web is used to aid delivery.^13^

Modes of delivery have also changed, with technological advances. Early e-therapy was sometimes packaged on CD-ROM and operated in a ‘stand-alone’ fashion on a PC, whereas practically all such tools are now accessed in one of two forms: as a web-based application (‘web app’), accessed via a conventional web browser, or else as a smartphone/tablet app, installed on (typically) the service user's mobile device. The distinction is somewhat arbitrary, but since smartphone apps represent a relatively more recent development in the digital domain, and a significant one too, in terms of popular uptake, it is convenient for this paper to consider e-therapy as divided into two main categories: *web apps* and *smartphone apps*.

### Policy history

The National Institute for Health and Care Excellence (NICE) is a non-departmental public body, responsible to but operationally independent of the UK Department of Health. Its function is to provide guidance to the NHS in England (although its advice often extends to the other constituent nations of the UK) for clinical practice, including what treatments should be offered for diseases, on the basis of published evidence. This remit includes the use of health technologies for mental ill health. NICE recommendations stand until they are revised or replaced. In 2006, NICE issued its first specific guidelines for e-therapy, recommending two computerised cognitive–behaviour therapy (cCBT) web apps for the treatment of mild to moderate depression and for panic/phobia, for which it was deemed there was sufficient evidence of clinical effectiveness. In 2009, these specific recommendations were withdrawn by NICE. At the time of writing (August 2016), NICE guidance for mental health practitioners is that cCBT can be offered for persistent subthreshold, or mild to moderate depression;^14^ however, reference to specific tools (with published evidence) has been replaced by general guidelines for cCBT.^14^ ^15^ CCBT is recommended for research purposes only for generalised anxiety disorder (GAD)^16^ and is not recommended at all for adult phobias.^17^

#### Recent technological developments

Since the first NICE recommendations for e-therapies,^14^ the use of smartphone and tablet computer has fundamentally altered the way that people interact with technology. On these devices, a plethora of health-related and mental health-related apps are available at very little or no cost to the user. However, the quality and effectiveness of these apps is often questionable, with no general requirement to demonstrate beneficial outcomes through clinical trials or other means. While recent policy changes mean that currently, some stand-alone software including smartphone apps installed onto a device for a medical purpose are now considered a ‘medical device’^18^ ^19^ and must be registered with the Medicines and Healthcare Products Regulatory Agency (MHRA), registration is not in itself an indication of efficacy.^20^

Meanwhile, the next generation of web apps includes features such as social networking which can lead to complex and dynamic interactions among users and technology. Unfortunately, the pace of change in smartphone and web health app development frequently renders the research community unable to evaluate programs fast enough to endorse or reject new interventions on the basis of evidence as potentially effective components in routine care. This shifting policy and technological landscape means that consulting NICE guidelines is no longer an effective way to find out which e-therapies are being routinely used and recommended across the NHS in England.

### Access to digital mental healthcare in the NHS in England

Understanding the digital mental health service landscape requires consideration of the methods of access to NHS-recommended digital healthcare in England. There are several points of access including referral and self-help routes.

#### Referral

##### Improving Access to Psychological Therapies

Much of the primary mental healthcare provision in the NHS in England currently comes through Improving Access to Psychological Therapies (IAPT) programme. IAPT was launched in 2007 to improve access to NICE-recommended psychological therapies for depression and anxiety disorders.^21^ IAPT services are provided on a local basis, sometimes alongside other health services, and offer direct routes to assessment and treatment by specialist mental health professionals without the need for general practitioner referral.

Owing to current NICE guidelines making general, rather than specific recommendations regarding e-therapies, practitioners in IAPT services are free to judge which apps are appropriate to use. Consequently, it is unclear which e-therapies are currently being recommended to and used by clients. Since mental health services in England are no longer exclusively provided by the NHS—charities, social enterprises, non-profit and limited companies can also provide IAPT services—variation compounds this lack of clarity.

##### NHS Mental Health Trusts

In addition, IAPT services can also be provided by Mental Health Trusts, which cater for severe mental health problems.^22^ In the same period in which rapid technical developments have fundamentally changed the way that people expect to access services in general, the NHS in England has undergone profound infrastructural changes in mental healthcare provision. Collectively, these factors make for a very unclear picture of what e-therapies are used and recommend by the NHS across England.

#### Self-help

In addition to accessing digital mental services via traditional face-to-face services (IAPTs or NHS Mental Health Trusts), there are also two avenues through which the NHS has sought to guide people's use of digital self-help for mental health concerns.

##### NHS Health Apps Library

In keeping with the NHS goals of becoming ‘more digitised’, and with providing service users with access to tools to support their own well-being, The NHS Commissioning Board launched the NHS Health Apps Library in March 2013.^23^ The library was a subsection of the NHS Choices website and provided a portal through which the public could access a selection of smartphone and tablet apps reviewed by the NHS. However, the library was shut down on 16 October 2015 after the publication of two papers that questioned the methods of evaluation of the apps recommended by the library. Specifically, the evaluation of apps' data security^24^ and clinical effectiveness^25^ were criticised.

##### NHS online Mental Health Apps Library

NHS Choices in March 2015 published a webpage entitled *Online Mental Health Services.*^26^ This page existed separately from the now-defunct NHS Health Apps Library, and, at the time of writing (August 2016), provides a list of six apps, all web apps, that have ‘been approved for use by the NHS’, although by whom and on what basis is unclear, and in fact seems to run counter to current NICE advice.

### Current study

Digital mental healthcare provision within the NHS in England is a diverse. Ever-evolving services provide different means of accessing digital healthcare products that are themselves the products of a highly dynamic marketplace, and with which official recommendations and advice struggles to keep pace. The key objective of this paper is to illuminate the current state of digital mental healthcare in England by documenting what e-therapies are used and recommended by the NHS, thus providing a starting point for evaluation of current practice.

## Methods

### Design

We documented web and smartphone apps used and recommended in the NHS for stress, anxiety and depression. Our data sources were fourfold. Using FOI requests, we requested a list of which web apps were being used and recommended in (1) NHS IAPT services and (2) NHS Mental Health Trusts. We also reviewed (3) the NHS Health Apps Library and (4) the NHS Mental Health Apps Library to identify apps (and web apps) that were currently (or recently) being endorsed by the NHS. In our FOIs to NHS IAPTs and Trusts, we also asked for information about involvement in research, piloting, or development of e-therapies, to capture the current practice, and insight into the slightly larger temporal window of very recent past, current, and likely future developments. All e-therapies were appraised against the inclusion criterion of being targeted to alleviate the symptoms of depression, anxiety or stress. To meet this criterion, the developer of the app had to be locatable via a Google search when entering the app name as the search term, and the app had to reference the targeted conditions in its marketing literature or be based on a therapeutic tool known to benefit the targeted conditions.

### Procedure

#### Improving Access to Psychological Therapies

On 10 February 2015, a list of IAPT services within England was requested through a FOI email to NHS Choices, asking for the contact details of all IAPT services within the country. This yielded a list of 295 IAPT services, of which only 116 were sufficiently detailed to identify their overarching IAPT service provider.^i^ Each service's provider was located via an internet search and overall, 111 IAPT service providers were identified.

On 13 February 2015, an FOI email request was sent to each of the 111 IAPT service providers. The questions asked are reported in online supplementary table S1. According to the FOI Act, requests must be answered within 20 working days of receipt. No responses were received after this time.

10.1136/bmjopen-2016-014844.supp1 supplementary table Freedom of Information Request Questions asked of IAPT Service Providers

#### NHS Mental Health Trusts

Many IAPT services are hosted by NHS Mental Health Trusts. It is possible that the answers given by IAPT services may be missing elements that are only be answerable at a NHS Trust level. For example, an IAPT service hosted by a Trust may not be aware of its host's activities around research and development. Therefore, FOI emails were also sent to each Trust, using a list of 51 NHS Mental Health Trusts compiled from the NHS Choices mental health trust listing page on 3 March 2015. The questions asked are reported in see online supplementary table S2. No responses were received after the mandated response window.

10.1136/bmjopen-2016-014844.supp2 supplementary table Freedom of Information Request Questions asked of NHS Trusts

#### NHS Apps Libraries

On 26 March 2015, web and smartphone apps were identified by carrying out four searches on the NHS Health Apps Library under the search terms ‘Mental Health’, ‘Depression’, ‘Anxiety’, ‘Stress’. Additionally, the apps listed when clicking on the navigation menu category ‘Mental Health’ were also collected. The apps listed on the NHS Mental Health Apps Library (on 2 November 2015) were also collected.

## Results

We present the data from each of the sources separately in the following sections. For IAPTs and trusts, we present data pertaining to: (1) response rates; (2) use of web and smart phone apps; (3) reports of being involved in research, piloting, or development of apps and (4) whether they support online self-referral (IAPTs only). For apps libraries, we report the apps which met our inclusion criterion. The final list of e-therapies reported as being used or recommended by IAPTs or Trusts in England, or listed on the NHS Apps libraries for common mental health problems, is summarised in table 1.

**Table 1:** All web and smartphone apps reported to be used or recommended by the NHS for common mental health problems

App	Format	Payment model	Web or phone based (w/p)	Number of IAPTs using/recommending (% of 191 IAPT services)	Number of Trusts using/recommending (% of 51 Trusts)	Listed in NHS Health Apps Library (y/n)	Listed in Mental Health Apps Library (y/n)
Living Life to the Full	Online modular self-help	Free to access	w	94 (49.2%)	24 (47.1%)	n	n
MoodGYM	Online modular self-help	Free to access	w	46 (24.1%)	10 (19.6%)	n	n
Big White Wall	Online forum with tools, courses and one-to-one messenger based chat with a professional.	Paid for by provider (but only available in some areas) or end user	w	39 (20.4%)	12 (23.5%)	y	y
Beating the Blues*	Online modular self-help	Paid for by provider (but only available in some areas) or end user	w	34 (17.8%)	13 (25.5%)	n	n
Silvercloud health	Online modular self-help with therapist support	Paid for by provider (but only available in some areas)	w	27 (14.1%)	5 (9.8%)	n	y
Ieso Digital Health	Online one-to-one messenger-based chat with a professional	Paid for by provider (but only available in some areas)	w	22 (11.5%)	5 (9.8%)	y	y
Fear Fighter	Online modular self-help	Paid for by provider (but only available in some areas) or end user	w	20 (10.5%)	5 (9.8%)	n	y
Headspace	Meditation via app or online	Paid for by end user	p	11 (5.8%)	3 (5.9%)	n	n
Buddy App†	Tool to support face-to-face therapy	Paid for by provider	w	6 (3.1%)	2 (3.9%)	y	y
Don't Panic!	Self-help resources	Free to access	p	5 (2.6%)	1 (2.0%)	n	n
MyMoodTracker	Mood tracker	Paid for by end user	p	2 (1.0%)	1 (2.0%)	n	n
Mindfulness Bell	Meditation	Paid for by end user	p	2 (1.0%)	0 (0%)	n	n
Moodkit—Mood Improvement Tools	Tools to improve mood	Paid for by end user	p	2 (1.0%)	0 (0%)	y	n
Thought Diary Pro	Thought diary	Paid for by end user	p	2 (1.0%)	0 (0%)	n	n
WellMind	Tools to help with depression, stress, anxiety	Free to access	p	2 (1.0%)	1 (2.0%)	n	n
Moodometer	Tool to support face-to-face therapy	Free to access	p	2 (1.0%)	0 (0%)	n	n
Kooth	Online one-to-one messenger-based chat with a professional for children and young adults aged 11–19	Free to access (but only available in some areas)	w	2 (1.0%)	2 (3.9%)	n	y
CBTReferee	Journal to assist face-to-face CBT	Paid for by end user	p	1 (0.5%)	1 (2.0%)	n	n
iCBT	Tool for self-help using CBT	Paid for by end user	p	1 (0.5%)	0 (0%)	n	n
Thought Diary	Thought diary	Paid for by end user	p	1 (0.5%)	0 (0%)	n	n
Stay Alive	Tools to prevent suicide	Free to access	p	1 (0.5%)	1 (2.0%)	n	n
Take a break!	Meditation app	Free to access	p	1 (0.5%)	1 (2.0%)	n	n
Mindshift	Tools to help with anxiety	Free to access	p	1 (0.5%)	0 (0%)	n	n
Moodscope	Tool to monitor mood	Free to access, stepped payment	w	1 (0.5%)	1 (2.0%)	y	n
DigitalMeds	Binaural beat technology for meditation	Paid for by end user	p	0 (0%)	0 (0%)	y	n
How Are You App	Mood tracker	Paid for by end user	p	0 (0%)	0 (0%)	y	n
Mindfulness by Digipill	Meditation	Paid for by end user	p	0 (0%)	0 (0%)	y	n
Mindlogr	Video journal	Paid for by end user	p	0 (0%)	0 (0%)	y	n
Panic Attack Aid	Tools to help with panic attacks	Paid for by end user	p	0 (0%)	0 (0%)	y	n
Phobia Free	Augmented Reality (AR) for exposure treatment	Paid for by end user	p	0 (0%)	0 (0%)	y	n
Stress Management App	Tools to help with stress	Paid for by end user	p	0 (0%)	0 (0%)	y	n
WorkGuru	Tools to help with stress at work	Paid for by end user	w	0 (0%)	0 (0%)	y	n
Worry Watch	Journal for anxiety	Paid for by end user	p	0 (0%)	0 (0%)	y	n
MindEd	Online advice and support	Free to access	w	0 (0%)	1 (2.0%)	n	n
Puffell	Online advice and support	Free to access	w	0 (0%)	1 (2.0%)	n	n
Virtual Hope Box	Tools to compliment face-to-face	Free to access	p	0 (0%)	1 (2.0%)	n	n
Aventurine Mood Improver	Tool for self-help using CBT	Free to access	p	0 (0%)	0 (0%)	y	n
Black Rainbow	Advice and audio for relaxation	Free to access	p	0 (0%)	0 (0%)	y	n
Depression Calculator	PHQ-9 screening tool	Free to access	p	0 (0%)	0 (0%)	y	n
Five Ways to Well-being	Tools for well-being	Free to access	p	0 (0%)	0 (0%)	y	n
Ginsberg	Activity and mood diary	Free to access	p	0 (0%)	0 (0%)	y	n
Happy Healthy App	Tools for well-being	Free to access	p	0 (0%)	0 (0%)	y	n
Healthstored	Health tracker	Free to access	p	0 (0%)	0 (0%)	y	n
Healthy Living	Guide to healthy living	Free to access	p	0 (0%)	0 (0%)	y	n
Hello Brain Health	Brain exercises for better health	Free to access	p	0 (0%)	0 (0%)	y	n
Moodbug	Mood tracker	Free to access	p	0 (0%)	0 (0%)	y	n
SAM: Self-help for Anxiety	Tools to help with anxiety	Free to access	p	0 (0%)	0 (0%)	y	n
Stress & Anxiety Companion	Tools to help with anxiety	Free to access	p	0 (0%)	0 (0%)	y	n

*Beating the Blues developer Ultrasis went into administration in October 2015. The program is now linked with 365 Health and Well-being who have been unreachable for comment.

†Buddy Enterprises has ceased operations and as a result of this Buddy App has been discontinued.

CBT, cognitive–behaviour therapy; IAPT, Improving Access to Psychological Therapies; NHS, National Health Service; PHQ-9, Patient Health Questionnaire.

### Improving Access to Psychological Therapies services

The FOI responses from IAPT services were inconsistent. Some providers answered at an overarching provider level while others gave granular detail regarding each of their service locations. In our results, we assumed that when a provider responded at top level that they referred to all their IAPT service locations. A total of 61 out of 111 IAPT service providers responded, accounting for 191 IAPT services. Two providers, one a charity and the other a Community Interest Company (CIC—a UK limited company whose objective is to benefit the community it serves, using any profits and assets for that purpose), refused to respond to the FOI on the grounds that the act did not apply to them; a further two acknowledged receiving the FOI request but did not follow up with a response to the questions asked, and 13 indicated that their services had been discontinued, merged with, or passed to another IAPT provider. A total of 33 IAPT providers did not respond at all to the FOI request. These comprised public sector organisations: n=8 (24.2%); third sector organisations: n=13 (39.4%); and private sector organisations: n=12 (36.4%). The majority of the non-responders were non-public sector organisations (n=25; 75.8%).

**Figure 1:**
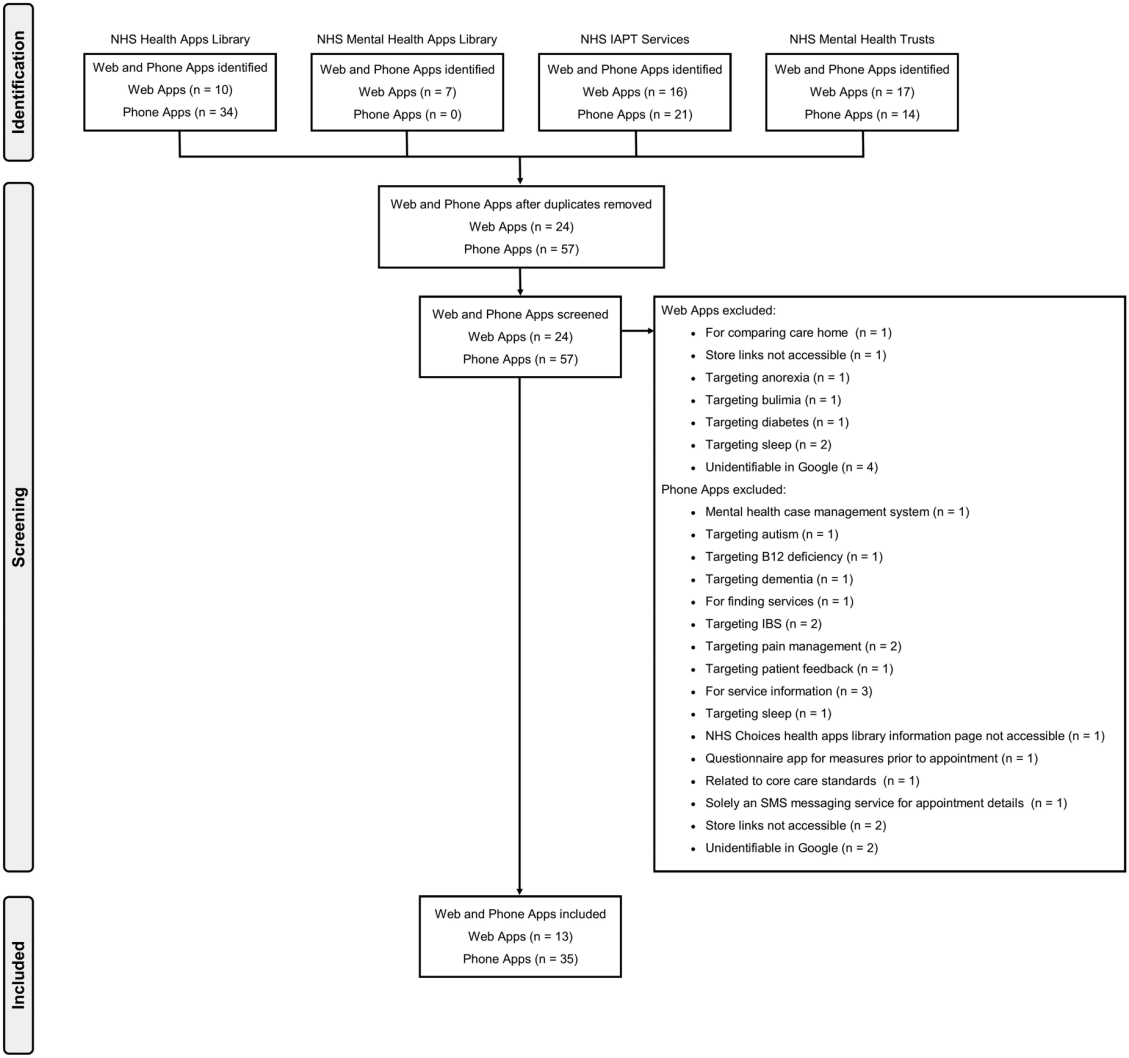
PRISMA flow diagram of app data collection. PRISMA, Preferred Reporting Items for Systematic review and Meta-Analysis.

One hundred and sixty-nine of the 191 (88.5%) IAPT services for which responses were obtained recommend or used web apps and of those, 41 (24.3%) use at least one of the NICE-recommended^27^ cCBT programs. IAPT services, in addition, highlighted 16 different web apps. Six were excluded for not meeting the inclusion criterion (figure 1) leaving 10 included web apps (table 1). Fifty (26.2%) of the IAPT services recommend or use smartphone apps, and 21 smartphone apps were specifically named. Seven did not meet the inclusion criteria (figure 1), leaving 14 included smartphone apps.

Twelve IAPT services indicated they were carrying out research into web apps, 10 stated they were piloting web apps and two said that they were in the process of developing their own. Regarding smartphone apps, 15 IAPT services indicated they were carrying out research into smartphone apps, two stated they were piloting smartphone apps and 10 said that they were in the process of developing their own. Two IAPTs indicated using patient webinars but did not disclose details of their content. Regarding online self-referral, 138 (72.3%) of the 191 IAPT services support this, either via email or online form.

### NHS Mental Health Trusts

All 51 Mental Health Trusts responded to the FOI request. Thirty-nine of the 51 (76.5%) trusts recommend web apps and of these, 14 (35.9%) use NICE-recommended cCBT.^28^ Seventeen web apps were highlighted by Trusts, five of which did not meet the inclusion criterion (figure 1). This left 12 included web apps (table 1). Fifteen (29.4%) of the 51 trusts recommend or use smartphone apps. Trusts named 14 specific smartphone apps, six of which did not meet the inclusion criterion (figure 1), leaving eight included smartphone apps (table 1).

One trust indicated it was carrying out research into web apps, two stated they were piloting web apps and one said that it was in the process of developing its own. Regarding smart phone apps, two Trusts indicated they were carrying out research into smartphone apps, two indicated they were piloting smartphone apps and seven said they were in the process of developing their own.

### Apps libraries

In the NHS Health Apps Library, a list of 44 web/smartphone apps were identified, 18 of these did not meet the inclusion criterion (figure 1) leaving 26 included apps. Out of the seven web apps listed in the NHS Mental Health Apps Library on 2 November 2015, six met the inclusion criterion and one did not (figure 1). Only three apps were present in both libraries (table 1).

## Discussion

The present paper is the first attempt to document all the e-therapies used and recommended by the NHS in England at a particular window in time. While the list of e-therapies is changeable over time, the present paper provides future researchers, commissioners and policymakers with a baseline of information from which to build. The data presented raise several interesting issues relating to the accessibility of service information, NICE guidelines on e-therapies, and ways of evaluating e-therapies.

### Data accessibility and quality

This study relied heavily on the provisions of the UK FOI Act (2000) for the collection of data. The reorganisation of the provision of mental health services in England has led to the increased participation of private and third sector provider organisations. Unlike public bodies, these organisations are not obliged to respond to FOI requests. Indeed, over half of the organisations that did not respond to the FOI request were limited companies or charities. As more areas of the NHS are outsourced to external providers, inaccessibility of service information is likely to increase. There is therefore a need for the FOI Act to apply to all NHS services, be they publicly or privately run, to ensure a level of transparency that allows positive and negative aspects of services to be made visible to the public and researchers alike.

Regarding the quality of the data collected, in certain cases, the FOI requests were answered with data sets that contained missing or inaccurate data. For instance, in the IAPT data set supplied by NHS Choices, which is searched by service users of the NHS Choices website, only 46.4% of services had provided a contact email and only 52.5% had a website link detailing more information about the service location. There is a clear need for the NHS to improve its data curation procedures to meet the aspiration of becoming truly digitally enabled.

### NICE guidelines on e-therapies

NICE (2006) recommended two cCBT programs for use within NHS services. The publication of subsequent NICE recommendations (2009b) resulted in the withdrawal of endorsement of any specific app, shifting responsibility for choosing e-therapies to service providers. Owing to this, there are now 13 different web apps, and 35 smart phone apps, for depression, anxiety or stress, available either through referral services or the NHS Mental Health Apps Library. These e-therapies are not consistently used or recommended across the country representing variability in service provision by geographical location. There are also notable differences between the four most used apps by IAPTs and Trusts, and the apps currently listed in the Mental Health Apps Library and previously listed in the Health Apps Library, with three of the four most used by IAPTs and Trusts not appearing in either library. Most notably, the top two apps used by IAPTs and Trusts are free to access, and yet are not listed in the current (or previous) NHS library. Perhaps this indicates different decision processes being used by IAPTs and Trusts compared with NHS library curators. Additionally, the current Mental Health Apps Library features many apps that are only free in some areas of England, requiring user payment in others. This has implications for patient choice and service equality. Furthermore, 11.5% of IAPT services and 23.5% of Trusts do not use or recommend web apps at all. It is not clear whether this reflects the absence of specific NICE guidelines, or a general lack of digitally enabled service provision.

### Ways of evaluating e-therapies

To help address the gap in NICE guidelines, it is crucial to investigate whether the apps currently being used are effective. While the pace of large scale evaluative research (eg, RCTs) lags behind that of advancing technology, there are other, more practical options for collecting and synthesising useful data. We make two specific recommendations. First, the minimal data set collected by IAPT^29^ which is used to build a picture of the current activity within IAPT services such as assessments, sessions, scales, should be revised. It currently requires data on whether a client is using an e-therapy, but it does not indicate which one.^30^ A more fine-grained approach, where e-therapy use, and which one, was recorded, would provide the ability to isolate the impact of individual apps on end users. This relatively small change to routine data collection practices would provide an instant evidence base against which all the e-therapies listed in the present paper could be evaluated. Second, for each e-therapy listed in the present paper, a systematic literature review should be conducted, to synthesise any existing effectiveness data.

There are also alternative methods of evaluating e-therapies, which, while they do not address effectiveness specifically, can provide useful insight into the integrity of the content, data security measures, and the acceptability to end users. Researchers have begun to discuss and propose methods of evaluating e-therapies. MindTech Framework for Mental Health Digital Products^31^ aids users in the process of evaluating, comparing and contrasting programs by providing a list of all possible and relevant issues. The Mobile Application Rating Scale (MARS)^32^ enables expert raters to review apps for engagement, functionality, visual aesthetics, information quality and subject quality of health apps and has been tested on Mindfulness apps.^33^ Other methods of analysis and evaluation might include syphoning review data from the app stores. While it cannot speak to effectiveness, rating scores and download numbers may give indications about acceptability.

Presently the NHS does not have a process in place to endorse apps. However, The National Information Board is working to develop a health app assessment process.^34^ This process will eventually enable the NHS to endorse apps. However, the consequences of endorsement are currently unknown. Endorsement may result in market dominance by those gaining NHS approval, stunting the market and truncating innovation.

### E-therapy tailored to specific demographics

The majority of the web/phone applications included in this review were not tailored to a specific demographic. One e-therapy Kooth was designed for young people aged 11–19. There were no e-therapies found for older adults aged over 65. It may be possible that some of the modular based e-therapies listed have the potential to support these groups through customised modules. It would be useful for future research to collect data on specific provision for different demographics, in terms of e-therapies available, and those employed by NHS services for specific demographics (eg, Child and Adolescent Mental Health Services).

## Conclusions

As e-therapies are continually evolving, their place within NHS services will also continue to change. However, there is a pressing need for proper evaluation of the effectiveness of the e-therapies used and recommended by the NHS, to support evidence-based practice, and help to overcome the gaps remaining in the NICE guidelines on apps for common mental health problems. The present paper has provided a starting point for this work, by documenting all the web-based and smartphone-based apps currently being used or recommended by the NHS in England. Future research should seek to examine the e-therapies identified within this paper and systematically review them for their clinical effectiveness. It is also important that changes are made to (1) enable better reporting of digital mental health service provisions within IAPT services, and (2) build an evidence base with which to evaluate the effectiveness of different e-therapies.

## Footnotes

i Two hundred and seventy-seven had websites. Some records had a generic website link to the general IAPT website (n=122), while others had addresses that displayed ‘no page found’ (n=31) or failed to reach any web page at all (n=7). This left 117 out of 277 IAPT services with a valid web address.

## References

[1] Dutton P Australia's first digital hospital 2014 http://www.health.gov.au/internet/ministers/publishing.nsf/Content/health-mediarel-yr2014-dutton109.htm (accessed 9 Sep 2016).

[2] Andersen AJW, Svensson T Internet-based mental health services in Norway and Sweden: characteristics and consequences. Adm Policy Ment Heal Ment Heal Serv Res 2013;40:145–53. 10.1007/s10488-011-0388-2 22109838

[3] National Information Board. Personalised health and care 2020, using data and technology to transform outcomes for patients and citizens, a framework for action 2014 https://www.gov.uk/government/uploads/system/uploads/attachment_data/file/384650/NIB_Report.pdf

[4] NHS England. Five year forward view 2014 https://www.england.nhs.uk/wp-content/uploads/2014/10/5yfv-web.pdf (accessed 9 Sep 2016).

[5] We Need to Talk coalition. We still need to talk: a report on access to talking therapies 2013.

[6] Mental Health Network. The future's digital mental health and technology 2014 http://www.nhsconfed.org/~/media/Confederation/Files/Publications/Documents/the-futures-digital.pdf

[7] Marks IM, Mataix-Cols D, Kenwright M Pragmatic evaluation of computer-aided self-help for anxiety and depression. Br J Psychiatry 2003;183:57–65. 10.1192/bjp.183.1.57 12835245

[8] Cuijpers P, Riper H Internet interventions for depressive disorders: an overview. Rev Psicopatología y Psicol Clínica 2014;19:209–16. 10.5944/rppc.vol.19.num.3.2014.13902

[9] Titov N Status of computerized cognitive behavioural therapy for adults. Aust N Z J Psychiatry 2007;41:95–114. 10.1080/00048670601109873 17464688

[10] Spurgeon JA, Wright JH Computer-assisted cognitive-behavioral therapy. Curr Psychiatry Rep 2010;12:547–52. 10.1007/s11920-010-0152-4 20872100

[11] Riper H, Andersson G, Christensen H Theme issue on e-mental health: a growing field in internet research. J Med Internet Res 2010;12:e74 10.2196/jmir.1713 21169177 PMC3057318

[12] Newman MG, Szkodny LE, Llera SJ A review of technology-assisted self-help and minimal contact therapies for drug and alcohol abuse and smoking addiction: Is human contact necessary for therapeutic efficacy? Clin Psychol Rev 2011;31:178–86. 10.1016/j.cpr.2010.10.002 21095051

[13] Barak A, Klein B, Proudfoot JG Defining internet-supported therapeutic interventions. Ann Behav Med 2009;38:4–17. 10.1007/s12160-009-9130-7 19787305

[14] National Institute for Health and Care Excellence. Depression in adults: recognition and management. CG90 2009:63 https://www.nice.org.uk/guidance/cg90/ (accessed 9 Sep 2016). 28230956

[15] NICE. Depression in adults with chronic physical health problem: recognition and management. CG91 2009.

[16] National Institute for Health and Care Excellence. Generalised anxiety disorder and panic disorder in adults: management. CG113 2011 https://www.nice.org.uk/guidance/cg113/ (accessed 9 Sep 2016).

[17] National Institute for Health and Care Excellence. Social anxiety disorder: Recognition, assessment and treatment. CG159 2013 https://www.nice.org.uk/guidance/cg159/ (accessed 9 Sep 2016).

[18] European Commission. Guidance document medical devices—scope, field of application, definition—qualification and classification of stand-alone software—MEDDEV 2.1/6 2016 http://ec.europa.eu/DocsRoom/documents/17921/attachments/1/translations/en/renditions/pdf

[19] Medicines & Healthcare products Regulatory Agency. Medical device stand-alone software including apps 2014 https://www.gov.uk/government/uploads/system/uploads/attachment_data/file/549127/Software_flow_chart_Ed_1-01.pdf (accessed 9 Sep 2016).

[20] McCartney M How do we know whether medical apps work? BMJ 2013;346:f1811 10.1136/bmj.f1811 23516158

[21] Clark DM Implementing NICE guidelines for the psychological treatment of depression and anxiety disorders: the IAPT experience. Int Rev Psychiatry 2011;23:318–27. 10.3109/09540261.2011.606803 22026487 PMC3212920

[22] NHS Choices. Authorities and trusts—The NHS in England—NHS Choices 2015 http://www.nhs.uk/NHSEngland/thenhs/about/Pages/authoritiesandtrusts.aspx (accessed 9 Sep 2016).

[23] NHS England. NHS Commissioning Board launches library of NHS-reviewed phone apps to help keep people healthy 2013 http://www.england.nhs.uk/2013/03/12/nhs-apps/ (accessed 9 Sep 2016).

[24] Huckvale K, Prieto JT, Tilney M Unaddressed privacy risks in accredited health and wellness apps: a cross-sectional systematic assessment. BMC Med 2015;13:214 10.1186/s12916-015-0444-y 26404673 PMC4582624

[25] Leigh S, Flatt S App-based psychological interventions: friend or foe ? Evid Based Ment Health 2015;18:97–9. 26459466 10.1136/eb-2015-102203 PMC11234577

[26] NHS Choices. Online mental health services 2015 http://www.nhs.uk/Conditions/online-mental-health-services/Pages/introduction.aspx (accessed 9 Sep 2016).

[27] National Institute for Health and Care Excellence. Computerised cognitive behaviour therapy for depression and anxiety. TA97 2006 https://www.nice.org.uk/guidance/ta97/ (accessed 9 Sep 2016).

[28] National Institute for Health and Care Excellence. Computerised cognitive behaviour therapy for depression and anxiety: review of technology appraisal 51 2008 http://www.nice.org.uk/guidance/ta97 (accessed 10 Dec 2014).

[29] NHS Digital. Improving access to psychological therapies data set 2016 http://content.digital.nhs.uk/iapt (accessed 9 Sep 2016).

[30] Health and Social Care Information Centre. Psychological therapies: annual report on the use of IAPT services England, 2014/15 2015 http://www.hscic.gov.uk/catalogue/PUB14899/psyc-ther-ann-rep-2013-14.pdf

[31] MindTech. Digital mental health toolkit—MindTech 2014 http://www.mindtech.org.uk/digital-mental-health-toolkit.html (accessed 20 Oct 2015).

[32] Stoyanov SR, Hides L, Kavanagh DJ Mobile app rating scale: a new tool for assessing the quality of health mobile apps. JMIR mHealth uHealth 2015;3:e27 10.2196/mhealth.3422 25760773 PMC4376132

[33] Mani M, Kavanagh DJ, Hides L Review and evaluation of mindfulness-based iPhone apps. JMIR mHealth uHealth 2015;3:e82 10.2196/mhealth.4328 26290327 PMC4705029

[34] National Information Board. WORK STREAM 1.2 ROADMAP. Enable me to make the right health and care choices. Providing citizens with access to an assessed set of NHS and social care ‘apps’ Work 2015 https://www.gov.uk/government/uploads/system/uploads/attachment_data/file/442830/Work_Stream_3.pdf (accessed 24 Nov 2016).

