# Supplementary material for: E-therapies in England for stress, anxiety or depression: what is being used in the NHS? A survey of mental health services
Source: BMJ Open. 2017 Jan 23;7(1):e014844. doi: 10.1136/bmjopen-2016-014844 (PMC5278266; doi:10.1136/bmjopen-2016-014844)
Supplement: supplementary table — Freedom of Information Request Questions asked of IAPT Service Providers [file bmjopen-2016-014844supp_tableS1.pdf]

Table S1. Freedom of Information Request Questions asked of IAPT Service Providers

Question	Wording
1.	According to the dataset I have obtained from NHS Choices, you host the following services [service names]. Please can you confirm whether this list is complete and add any additional services that are not listed? (Including address and postcode).
3.	If these services are not run directly by the NHS please state who runs them and what they are (e.g. Social Enterprise, Limited Company, third sector group).
4.	If you provide an IAPT a service on behalf of another organisation (such as a care commissioning group, foundation trust or other) please state the organisation's name.
5.	Please state the types of referral used by your organisation / IAPT i.e. GP, Self-Referral, Other.
6.	If you offer a self-referral service do you have an online self-referral method such as a contact email address or a form on your website?
7.	Please state any e-therapies, computerised therapies, Internet-delivered therapies, online therapies, or advice/guidance websites for mental health issues that your organisation / IAPT service use or recommend to service users. For example, these might include, but are not limited to internet-delivered cognitive behaviour therapy with or without therapist support, internet-delivered therapy

based on an approach other than cognitive behaviour therapy, online or email therapy/counselling, online social support networks, or informational websites.

8. Please state any smart phone apps your organisation / IAPT service use or recommend to service users. For example, these might include specific apps from the NHS Choices Health Apps Library, or others that professionals in your service recommend.

9. In 2013/14 could you give the total number of people your service saw under IAPT and also give a figure as to how many of those were aged 50 or over.
