# Supplementary material for: E-therapies in England for stress, anxiety or depression: what is being used in the NHS? A survey of mental health services
Source: BMJ Open. 2017 Jan 23;7(1):e014844. doi: 10.1136/bmjopen-2016-014844 (PMC5278266; doi:10.1136/bmjopen-2016-014844)
Supplement: supplementary table — Freedom of Information Request Questions asked of NHS Trusts [file bmjopen-2016-014844supp_tableS2.pdf]

Table S2. Freedom of Information Request Questions asked of NHS Trusts

Question	Wording
1.	Please state any e-therapies, computerised therapies, Internet-delivered therapies, online therapies, or advice/guidance websites for mental health issues that your trust use or recommend to service users. For example, these might include, but are not limited to internet-delivered cognitive behaviour therapy with or without therapist support, internet-delivered therapy based on an approach other than cognitive behaviour therapy, online or email therapy/counselling, online social support networks, or informational websites. Format this as a list indicating which are recommended and which are used by the trust.
2.	Please state any smart phone apps your trust use or recommend to service users. For example, these might include specific apps from the NHS Choices Health Apps Library, or others that professionals in your service recommend. Format this as a list indicating which are recommended and which are used by the trust.
3.	For the period 2013/14 could you state the total number of people your trust treated for depression / anxiety and also give a figure as to how many of those were aged 65 or over.
